# Molecular Cloning, Expression, Purification, and Functional Characterization of Dammarenediol Synthase from *Panax ginseng*

**DOI:** 10.1155/2013/285740

**Published:** 2012-12-30

**Authors:** Wei Hu, Ning Liu, Yuhua Tian, Lianxue Zhang

**Affiliations:** ^1^College of Life Science, Jilin Agriculture University, Changchun 130118, China; ^2^College of Traditional Chinese Medicine, Jilin Agriculture University, Changchun 130118, China

## Abstract

The objective of this study is to clone and charecterize the expression of dammarenediol synthase gene and then to determine the relationship between the expression of dammarenediol synthase gene that is involved in the ginsenoside biosynthetic pathway and the ginsenoside content. A cDNA phage library was constructed from a five-year-old ginseng root. The cDNA library was screened for the dammarenediol synthase gene by using its specific primers. It was further cloned and expressed in pET-30a vector. The recombinant plasmid pET-30a-DS was expressed in Rosetta *E. coli*. The recombinant DS protein was purified by affinity chromatography. The production of dammarenediol was detected by liquid chromatography-mass spectrometry (LC-MS). Results showed that dammarenediol synthase gene was cloned from the cDNA library and was expressed in Rosetta *E. coli* and the SDS-PAGE analysis showed the presence of purified DS protein. LS-MS showed the activity of DS protein, as the protein content increases the dammarenediol increases. Our results indicate that the recombinant dammarenediol synthase protein could increase the production of dammarenediol and the expression of DS played a vital role in the biosynthesis of ginsenosides in *P. ginseng*.

## 1. Introduction 

Medicinal *Panax ginseng C. A. Meyer* belongs to *Panax* genus under Araliaceae family. As a traditional medicinal plant, it has been in use for thousands of years. Modern medicinal chemistry and pharmacology studies have confirmed that various compositions of ginseng have a role in enhancing the immunity, neural protection, antiaging, antidepressant, antitumor, and other aspects [1–6] and also have good application prospect with considerable economic value. 

In spite of these prospects, the difficulty of ginsenosides supplies in pure form at larger quantities has prevented in usage of them as clinical drugs. And ginsengs' quality and growth was severely restricted by its low growth rate, due to the impact of climate, geography, and cultivation conditions [7, 8]. Isolation and purification of each ginsenoside involves longer and complicated procedures. Alternatively, the expression of the genes for biosynthetic enzymes in heterologous host organisms, hopefully in fast growing microbes such as *Escherichia coli*, can lead to ginsenoside production in larger quantities. Genetic engineering was expected to become the most promising way to produce medicinal secondary metabolites of low content, and cloning of the relevant genes of ginsenoside biosynthesis is highly expected. 

Ginsenoside belongs to triterpenoid, which could be classified into dammarane type and oleanane type [9, 10] according to the basic structure of aglycone, and most of the ginsenoside were of dammarane type. At present, the biosynthetic process can be divided into 3 processes: (1) synthesis of 3 isoprene pyrophosphate and two methyl allyl pyrophosphate; (2) synthesis of important intermediate precursors of 2,3-squalene oxide; and (3) cyclization of 2,3 oxidation of squalene [10, 11]. The cyclization of oxidosqualene is a biosynthetic branching which directs not only for phytosterols and triterpenes, but also for dammarane and oleanane type ginsenosides. In previous study, the first committed step in ginsenoside synthesis is the cyclization of 2, 3-oxidosqualene to dammarenediol II, catalyzed by the DS of the oxidosqualene cyclase group [12]. It means that the oxidation of squalene was first synthesized into dammarenediol under the action of the dammarenediol synthase (DS), and then generated to be various types of dammarane saponins through a series of hydroxylation and glycosylation reactions [10].

In the recent years, many researches focus on developing effective biological systems for the ginsenoside production by using recombinant technology. Recently, a large number of pentacyclic triterpene synthases have been cloned and functionally characterized from various plant species [13–15]. However, the expression of the genes for biosynthetic enzymes in heterologous host organisms, such as *Escherichia coli*, has not been obtained. And whether there was a certain correlation of the DS activity and saponin content *in vitro* was still in need of experimental verification. In our present study, we attempted to clone and express the dammarenediol synthase in prokaryotic system, the first committed enzyme for the biosynthesis of genuine sapogenin of dammarane type ginsenosides. It was shown that endogenous dammarenediol synthase protein was obtained by prokaryotic expression and analyzed the correlation of the DS activity and the accumulation of dammarenediol by LC-MS.

## 2. Materials and Methods

### 2.1. Experimental Material

One-, three-, four-, and five-year-old ginseng were taken from the Medicinal Botanical Garden, Jilin Agricultural University, and the fibrous roots of ginsengs root tissues were used in experimental studies.

### 2.2. Reagents

Trizol reagent was purchased from Invitrogen, Smart TM cDNA Library Construction kit was purchased from Clontech company, USA, Gigapack*Ⅲ* Gold packing extract kit was purchased from the Stratagene company, Reverse transcriptase, Ex Taq DNA polymerase, pMD18-T vector, DL-2000 Marker were purchased from TaKaRa company, Real Master Mix was purchased from the Changchun company, Ni-agarose protein purification kit was purchased from the Beijing Kangwei Century Company, Squalene was purchased from the Changchun DingGuo Biotechnology Co., Ltd., Dammarenediol Standard was purchased from the YunNan XiLi Biotechnology Co., Ltd., Squalene epoxidase enzyme was prepared in the laboratory, and *E. coli* Strain Rosetta and pET-30a Expression Vector were from our laboratory.

### 2.3. Construction and Detection cDNA Phage Library of Ginseng Root Tissue

Total RNA from different year-old ginseng root tissues was extracted by using Trizol. cDNA was synthesized by using the total RNA as template, with AMV reverse transcriptase and oligo-dT primers. full-length cDNA library of five-year-old *P. ginseng* was constructed by using SmartTM cDNA Library Construction kit. selection of the plaque, the detection of the size of the inserted fragment, and calculation of the reorganization rate was performed by PCR (Sense: CCATTGTGTTGGTACC CGG, Antisense: ATACGACTCACTATAG GGCGAATT).

### 2.4. DS Gene Cloning and Sequence Analysis

DS gene conservative primers (Sense: ATTAAGAATGTGGAAGCAGAAGG,  Antisense: CTTAAATTTTGAGCTGCTGGTG) were designed based on the related sequences of GenBank and used to screen the DS gene from the cDNA library. The targeted PCR fragment was recovered after agarose gel electrophoresis and cloned into the pMD18-T vector. To identify the correct recombinant plasmid, it was sequenced (Shanghai Sheng Gong biological engineering, Shanghai), and the DS gene DNA sequences were submitted to GenBank (accession number: JN596111). The name of the correct construct is pMD-18T-DS.

### 2.5. Construction and Identification of pET-30a-DS

pET-30a and DS gene fragments from pMD-18T-DS plasmid were digested by *Nco*I and *Not*I, and ligated with T4DNA ligase at 16°C, then transformed into Rosetta *E. coli *competent cell, and cultured overnight at 37°C. Positive clones were selected by double digestion and to identify the correct insert, it was sent for sequencing. The positive clone was named as pET-30a-DS.

### 2.6. Induced Expression of Recombinant and Purification of Fusion Protein

*E. coli *cells harboring an expression construct were grown in 10 mL LB broth containing 30 *μ*g/mL kanamycin plus 34 *μ*g/mL chloramphenicol for Rosetta at 37°C overnight with constant shaking. The recombinant bacteria were inoculated in LB medium and cultured at 37°C to OD600 of 0.4 to 0.6, with different IPTG concentration (final concentrations of 0.1, 0.2, 0.6, 0.8, and 1.0 mmol/L) to induce expression, and induced for 4 h under the determined optimal concentration of IPTG. The bacterial lysate was collected 4 h later and detected by SDS-PAGE electrophoresis, with the noninduced recombinant Rosetta as the control.

Overnight cultures of bacterial cells were diluted 50-fold into 1 L LB broth, supplemented with appropriate antibiotics, cultured as above until an OD600 of 0.4–0.6 was reached, and then induced to express the recombinant proteins for 4 h by adding isopropyl **β**-D-galactopyranoside (IPTG) to a final concentration of 0.6 mM. The cells were sonicated for 2 min, and the precipitate was dissolved by Soluble Binding Buffer (20 mmol/L Tris-HCl, 10 mmol/L, imidazole, 0.5 mol/L NaCl) solution. The precipitate obtained from 12000 g centrifugation for 20 min is the inclusion body. Inclusion body was dissolved in ice water bath for 1 h and centrifuged at 10000 g for 20 min, and supernatant, were filtered by 0.45 *μ*m membrane filtration. After the balanced Ni-agarose affinity chromatography, the His tagged recombinant proteins were solubilized by Soluble Binding Buffer solvent and purified by Ni-NTA agarose chromatography; finally the elution peaks were collected according to 280 nm ultraviolet absorption value.

### 2.7. Detection of the Activity of the Recombinant Protein

In the present study, SEQ enzyme was expressed and purified using prokaryotic expression vector, which had been completed previously. The process of the catalytic reaction of DS enzyme involves two stages. Firstly, squalene was selected as a substrate. Under the same amount of substrate, squalene was catalyzed with the same SQE enzyme amount to produce the intermediate product of 2,3-oxidosqualene in different reactions systems. Subsequently, 2,3-oxidosqualene was further catalyzed with different DS enzyme amounts to produce dammarandiol. After the completion of enzymatic reaction, the enzyme was inactivated by heating at 100°C. Based on the characteristics of high solubility of the sapogenin dammarandiol in ethanol and low solubility in acetone, following extraction by ethanol, acetone was added into ethanol to separate out dammarandiol, which was then detected by LC-MS technique. 

The experiment could be divided into five DS enzyme reaction systems. Substrate squalene (20 L) were added into a centrifugal tube which have Tris-HCl buffer (pH 6.0) in it, and then added the DS enzyme solution (0.65 g/mL) 5 *μ*L, 10 *μ*L, 15 *μ*L, 20 *μ*L, 25 *μ*L respectively, and added SQE enzyme solution (0.35 g/mL) 20 *μ*L in each of the centrifugal tube and they reacted at 37°C for 1 h, and then the temperature was raised to 100°C. After 10 min, the enzymatic reaction was stopped. The dammarnediol production was detected by liquid chromatography-tandem mass spectrometry.

Chromatographic column condition: Zorbax Extend-C18 column, 5 *μ*m particle size, 150 × 4.6 mmL·D, American Agilent company; mobile phase: water, acetonitrile, formic acid; flow: 0.8 mL/min; column temperature: room temperature; sample size: 25 *μ*L. Mass spectrometric conditions: electrospray ionization source (ESI); ion injection voltage: 5500 V; temperature: 550°C; source gas 1 (GS1, N2) pressure: 70 psi; gas 2 (GS2, N2) pressure: 60 psi; positive ion mode monitoring: scanning mode for the positive ion monitoring; DP voltage: 70 V; EP voltage: 10 V; CXP: 4.0 V; disintegration energy: 40 V. The ion pair for the qualitative analysis is 391/93.1; the ion pair for quantitative analysis is 391/149. Products of different enzyme reaction systems are injected into the injection hole to observe the changes of elution peak area. The standard concentration of dammarenediol was 1 mg/L and the generation amount of dammarenediol was calculated by comparing with the peak area ratio of the standard.

## 3. Results and Analysis

### 3.1. Synthesis of Double-Strand cDNA and Quality Detection of Phage Library

After detecting ultraviolet spectrophotometry, the A260/A280 of total RNA is 1.96. The electrophoresis results showed that the double-stranded cDNA is located between 0.3 and 2.5 kb, presenting an elongated waterfall-like band, which agreed well with the distribution of plant tissue DNA. According to the number of bacterial colony and bacteria liquid PCR identification results, the recombination rate was 95.17%. The capacity of the original library is 8.38 × 10^6^ pfu/mL^−1^.

### 3.2. DS Gene Cloning and Sequence Analysis

After electrophoresis, the PCR amplification product of DS gene obtained a specific band about 2310 bp, which agreed well with the expected results. pMD-18T-DS plasmid was digested by *Xba*I and *Pst*I and an insert size of 2310 bp, the size which corresponds to the target strap was obtained. BLAST analysis found that the DS cDNA carried a 2310 bp full open reading frame (ORF) fragment. The amino acids of DS (769 amino acids with a predicted molecular mass of 84.6 kDa) were found to be 56, 50, and 48% identical to those of **β**-amyrin synthase, cycloartenol synthase, and lanosterol synthase in *P. ginseng*.

### 3.3. Identification of Recombinant Expression Vector and Induced Expression of Recombinant

Specific band with the size about 2310 bp, consistent with expectation, was obtained by the application of *Nco*I and *Not*I double digestion pET-30a-DS. The results found that DS gene was inserted into pET-30a vector, and the recombinant plasmid pET-30a-DS was constructed.

**Figure 1:**
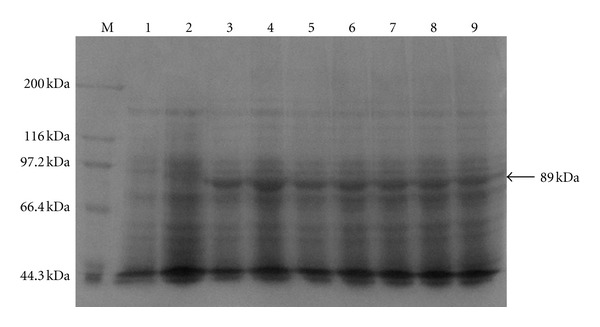
SDS-PAGE analysis of the DS expression in *E.coli*. Lane M: molecular weight marker; Lane 1: crude cells extracts of uninduced *E. coli* Rosetta containing pET-30a; Lane 2: crude cell extracts after 4 h past the induction without IPTG of *E. coli* Rosetta containing pET-30a-His-DS; Lane 3–9: crude cell extracts after 4 h past the induction with IPTG of *E. coli* Rosetta containing pET-30a-His-DS. The molecular weights of the new protein components agree well with those predicted for the fusion proteins.

**Figure 2:**
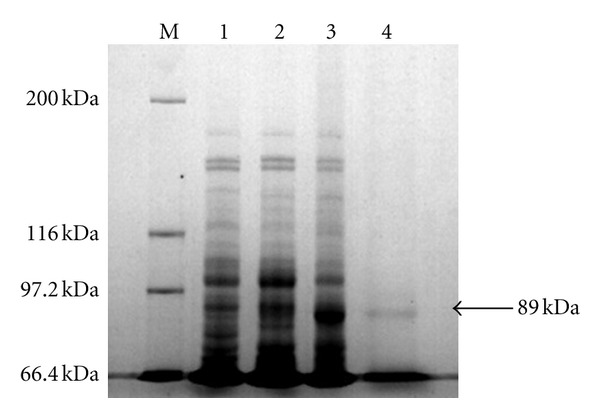
Purification result of recombinant protein. Lane Ms: protein molecular weight marker; Lane1: crude cell extracts of uninduced *E. coli* Rosetta containing pET-30a; Lane 2: crude cell extracts after 4 h past the induction without IPTG of *E. coli* Rosetta containing pET-30a-His-DS; Lane3: crude cell extracts after 4 h past the induction with IPTG of *E. coli* Rosetta containing pET-30a-His-DS. Lane4: the purified recombinant protein of DS. The purified molecular mass fused with His-tag protein agree well with those predicted for the fusion proteins.

 The recombinant plasmid pET-30a-DS was transformed into Rosetta *E. coli* to be induced for gene expression. The expressed product was identified by SDS-PAGE, and specific protein bands at about 89 kDa were found, which matched the molecular weight size of the predicted His tagged recombinant protein (Figure 1). When the IPTG final concentration reached to 0.6 mmol/L, protein expression is highest, and the difference of protein expression was not significant at different time intervals (1, 2, 3, 4, 5, 6, and 7 h). Purification of recombinant proteins by SDS-PAGE analysis showed that there were protein bands at about 89 kDa, which had the same molecular weight with fusion protein pET-30a-DS, and highly purity (Figure 2).

### 3.4. Biological Activity Detection of Recombinant Proteins

**Figure 3:**
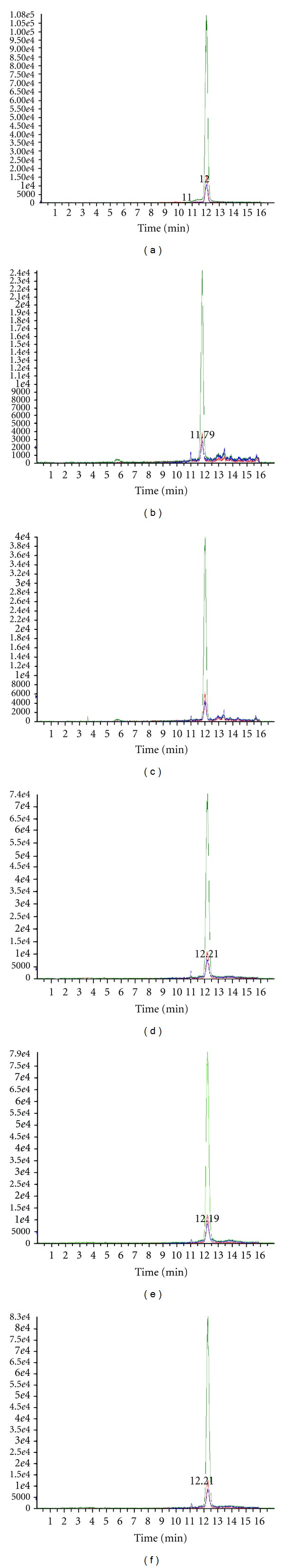
Extraction ion flow diagram of LC-MS mass spectrometer (indicates the peak). The horizontal coordinate and vertical coordinate indicates time of peak appearance and peak height. (a) the time of peak appearance of standard dammarendiol sample; (b)–(f) the time of peak appearance of dammarendiol in the reaction systems with different DS enzyme amounts (the DS enzyme amounts are 5, 10, 15, 20, and 25 *μ*L, resp.).

Dammarenediol was identified by the extraction ion chromatography of LC-MS and the retention time of peaks was 12.0 min. The LC-MS detection showed that under the condition of similar amounts of the substrate squalene, with the increase in the amount of DS enzyme, compared with the dammarandiol standard peak area, the generation amount of damarindiol gradually increased. The experimental results indicated that the recombinant proteins expressed in *E. coli* had the activity, and it could generate squalene into dammarenediol. At the same time, a close correlation of dammarenediol synthetase and the generation amount of ginsenoside were found (Figure 3 and Table 1).

## 4. Discussion

The cDNA library constructed by SMART technology ensured the integrity of cDNA library information [16, 17]. In addition, such kind of cDNA library construction of the five-year-old ginseng root was rarely reported. SMART technology can improve expression probability and recombination efficiency of the insertion region gene, so that the low-abundance genes in the library were enriched, which was in line with the low abundance of the gene expression in ginseng root separation. The original storage capacity of the five-year-old ginseng root cDNA library constructed in this study is greater than 10^6^ pfu/mL^−1^, inserted fragments were mainly concentrated between 500 and 2500 bp, which has reached the standard value of library and has laid the foundation for screening the key genes in the ginsenoside biosynthetic pathway.

The first committed step in ginsenoside synthesis is the cyclization of 2,3-oxidosqualene to dammarenediol, catalyzed by the DS of the oxidosqualene cyclase (OSC) group. Dammarenediol synthase is the most important enzyme in the biosynthetic pathway, which had a great relevance to the ginsenoside content. Additionally, the content was extremely low in ginseng root [18]. Elder ginseng from exuberant growth and metabolism season, with relative high expression of all types of genes were, chosen in this study. And the DS gene of open reading frame encoding 769 amino acids was achieved by the constructed cDNA library, thus a reference for further understanding of the DS gene characteristics and functions was provided. Cloning of dammarenediol synthase cDNA in this study has provided a significant step for the production of dammarane type ginsenosides by heterologous expression systems, since subsequent hydroxylation(s) and glycosylation(s) lead to a varieties of ginsenoside structures.

Recent years research on obtaining the active key enzyme protein by prokaryotic expression vector has rarely been reported. Morita et al. [15] have observed the production of dammarenediol II by the expression of PNA in an ERG7-deficient yeast-mutant (GIL77), but its regulatory mechanism is unclear. We inserted the ORF of DS cDNA in the pET-30a expression vector, and dammarenediol synthase was expressed and purified with high purity which is shown in SDS-PAGE. Because the DS gene sequence contained a large number of rare codons, the expression in *E. coli* Origami B (DE3) was lower and a series of Rosetta expression bacteria were used to help improve the expression quantity. The reason was that the PRARE2 plasmid was carried in Rosetta bacteria, which could complement tRNA corresponding to the seven rare codons absent from *E. coli*, and when compared with the Origami B (DE3) bacteria, it was more suitable for the expression of proteins containing a rare codon [19, 20].

Based on the above research, the dammarenediol generation amount in different amounts of DS enzyme reaction was analyzed by LC-MS technology. The LC/MS method had high specificity, sensitivity, accuracy, and other advantages, with the detection range of 2.0 ng·mL^−1^ [21, 22]. LC-MS analysis revealed that under the similar amount of the substrate squalene, with a corresponding decrease in the amount of DS enzyme, the generation amount of dammarenediol progressively increased, which illustrated that the recombinant protein expressed in *E. coli* had activity. The experiments further proved evidence to the close correlation of dammarenediol synthetase and saponin production *in vitro*, but the specific catalytic activity was not clear yet. Han et al. [23] studied on DS ginseng gene by RNA interference and found that RNA interference of DS led to a reduction of ginsenoside production to 84.5% in *P. ginseng* roots, which was very similar to the findings of this study. These results indicated that the overexpression of DS will be useful for the enhanced production of pharmacologically-important ginsenosides from *P. ginseng *or other plant species by genetic transformation.

In conclusion, this study explained further that DS gene plays a key role in ginsenoside biosynthesis pathway, ginsenoside accumulation was inseparable from the key genes expression levels changes, to a certain extent it could determine the trend of triterpene saponin biosynthesis, and DS was one of the key enzymes in control of ginsenoside synthesis. Therefore, to improve the expression of ginseng DS gene by the genetic engineering technology was most likely an effective means to increase the amount of ginsenosides. Therefore, our study provides a basic experimental data available for the study on DS genes and also provides a theoretical reference for the postdepth study of the DS gene function and regulatory mechanism on the biosynthetic pathway of ginsenoside.

## Figures and Tables

**Table 1:** Detection of dammarenediol with LC/MS method.

The amount of DS enzyme (*μ*L)	Peak area	Dammarenediol content (*μ*g)
0 (standard)	1.2847*e*5	1
5	2.8587*e*4	0.2225
10	4.9109*e*4	0.3823
15	6.3974*e*4	0.49810
20	9.1037*e*4	0.7086
25	1.1040*e*5	0.8593
